# Metabolic rewiring through succinate–GPR91 signaling: a fresh perspective on HFpEF energetics

**DOI:** 10.1186/s12933-026-03107-1

**Published:** 2026-03-23

**Authors:** Marialucia Telesca, Valeria Masciovecchio, Sarah Costantino

**Affiliations:** Center for Translational and Experimental Cardiology (CTEC), Department of Cardiology, University Hospital Zürich and University of Zürich, Wagistrasse 12, Schlieren, 8952 Switzerland

## Abstract

Succinate has recently emerged as a signaling metabolite that extends beyond its canonical role in the tricarboxylic acid (TCA) cycle to influence cellular adaptation and stress responses. In their study, Jia et al. identify the succinate-GPR91 axis as a key regulator of cardiomyocyte metabolic reprogramming and NAD^+^ homeostasis in heart failure with preserved ejection fraction (HFpEF). Their findings suggest that restoring succinate-GPR91 signaling enhances mitochondrial energetics, improves redox balance, and alleviates diastolic dysfunction. This commentary discusses the significance of these results in the broader context of cardiometabolic disease, highlighting the conceptual novelty of metabolic rewiring as a form of cardioprotection, while also addressing unresolved questions regarding tissue specificity, long-term signaling balance, and translational potential. In recent years, succinate has emerged as a multifaceted player not merely a tricarboxylic acid (TCA) cycle intermediate but also a stress‑responsive metabolite that conveys cellular metabolic state to neighbouring cells and distant tissues. It accumulates during ischemia, hypoxia, or mitochondrial dysfunction and can drive reverse electron transport at complex I, thereby increasing reactive oxygen species (ROS) production [1, 3]. This biochemical duality on the one hand enabling damaging ROS generation, and on the other acting extracellularly via the G protein-coupled receptor GPR91-raises a central question: is succinate–GPR91 signaling protective, maladaptive, or fundamentally context‑dependent? Demonstrating that succinate can act extracellularly through GPR91 to reprogram cardiac metabolism would recast it from a metabolic by‑product into a bona fide signaling molecule with therapeutic implications. In this issue, Jia et al.^2^ provide compelling evidence that the succinate-GPR91 axis functions as a molecular conduit linking mitochondrial metabolism to cardiomyocyte energy reprogramming, restoring NAD + and attenuating diastolic dysfunction in heart failure with preserved ejection fraction (HFpEF).

## Succinate signaling as a Bridge between metabolic rewiring and cardioprotection

Succinate occupies a central position in the TCA cycle and features prominently in the metabolic stress response. Transient activation of succinate-GPR91 can support acute metabolic adaptation; conversely, prolonged or dysregulated activation can be harmful. In ischemia-reperfusion (IR) injury, for example, succinate accumulation during ischemia drives a burst of ROS on reperfusion that initiates maladaptive cascades-ERK1/2-CaMKIIδ-HDAC5 hypertrophic signaling [1], mitochondrial fission, and cardiomyocyte apoptosis-contributing to ventricular remodeling and dysfunction. [4–6] Elevated circulating succinate has also been associated with adverse electrophysiologic outcomes, including increased atrial fibrillation in diet‑induced obesity and type‑2 diabetes models [6]. These findings have emphasized succinate’s injurious potential in acute settings.

Jia et al. [2] shift the focus to a chronic cardiometabolic HFpEF, where systemic metabolic dysfunction, rather than a single ischemic insult, predominates. Across human myocardial samples, multiple murine HFpEF models, and human cardiomyocyte-derived organoids, the authors report markedly reduced myocardial succinate levels and downregulated GPR91 expression. This surprising baseline deficit suggests that, in cardiometabolic HFpEF, succinate signaling is blunted rather than overactive. Using a “two‑hit” mouse model (high‑fat diet plus L‑NAME) that recapitulates the metabolic and diastolic features of cardiometabolic HFpEF [7], they show that exogenous succinate supplementation improves diastolic function, reduces fibrosis and hypertrophy, preserves mitochondrial ultrastructure, and improves systemic metabolic parameters. These effects indicate that succinate-GPR91 signaling can enable metabolic rescue in a chronic, multifactorial disease milieu where insulin resistance, lipotoxicity, altered TCA flux, and relative hypoxia intersect to shape cardiac remodeling.

## Cardiomyocyte GPR91 as a metabolic node and NAD^+^ as a downstream effector

A central strength of the study by Jia et al. [2] is the elegant genetic and mechanistic dissection showing that cardiomyocyte GPR91 functions as a critical metabolic sensor. Loss‑of‑function experiments-both global Gpr91^−/−^ and cardiomyocyte‑specific Gpr91ΔCM knockouts-abolished the protective effects of succinate, implicating cardiomyocyte GPR91 as a necessary transducer. Complementary in vitro studies using human pluripotent stem cell-derived cardiomyocytes and organoids corroborated the in vivo observations and permitted pathway analysis. Mechanistically, succinate-GPR91 signaling couples to Gq proteins and activates AMPK, a canonical regulator of cellular energy homeostasis. AMPK activation in turn promotes transcriptional upregulation of NAD+ biosynthetic pathways, most notably NAMPT, and represses the NAD+‑consuming enzyme CD38. The net effect is replenishment of intracellular NAD+ pools, restoration of redox balance, and improved metabolic flexibility. Given NAD+’s central role in sirtuin activity, PARP function, mitochondrial oxidative phosphorylation, and redox buffering [1, 3, 9], its restoration plausibly underlies much of the observed structural and functional myocardial benefit. Nonetheless, an important question remains unresolved: is NAD+ restoration the proximate mediator of cardioprotection, or does it merely mark upstream improvements in mitochondrial function? Determining causality whether NAD+ repletion alone can reproduce the benefits or whether it must occur in the context of succinate-GPR91-AMPK signaling is critical for prioritizing therapeutic strategies that target NAD+ metabolism versus receptor‑ or kinase‑level interventions.

### Succinate–GPR91: a multicellular signal in hfpef?

Modern HFpEF research increasingly recognizes the syndrome as a multisystem disorder driven by multi‑organ metabolic stress, endothelial dysfunction, and chronic low‑grade inflammation [7, 8]. Succinate signaling extends beyond cardiomyocytes: GPR91 is expressed on endothelial cells, macrophages, fibroblasts, and adipocytes, enabling succinate to orchestrate inter‑tissue communication. Jia et al.’s supplemental data expand the cardiac findings to a broader organismal level, showing that succinate-GPR91 signaling modulates adipose remodeling, systemic glucose and lipid metabolism, and whole‑body NAD+ balance. [2] This supports a model of HFpEF as a disorder of disrupted metabolic cross‑talk among adipose tissue, skeletal muscle, vasculature, immune cells, and myocardium [8–10]. This multicellular reach, however, introduces paradoxes. While transient succinate signaling may support cardiomyocyte adaptation, signaling in nonmyocyte compartments can be pro‑inflammatory and pro‑fibrotic, potentially driving adverse remodeling. Thus, systemic succinate augmentation risks beneficial myocardial effects being offset, or even outweighed, by maladaptive responses in endothelial cells, fibroblasts, or immune cells. These conflicting actions highlight the imperative to define tissue‑specific signaling consequences and to develop strategies that harness cardiomyocyte‑protective pathways without provoking deleterious systemic effects.

## Rewiring metabolism to redefine cardioprotection

The study by Jia et al. [2] reframes HFpEF through a metabolic lens, positioning the succinate-GPR91-AMPK-NAD+ axis as a unifying pathway that links mitochondrial energetics to cardiac function. Their findings support a model in which chronic metabolic disturbances, not solely hemodynamic overload, drive diastolic dysfunction, and in which restoring metabolic signaling can provide functional rescue. Translationally, this axis presents multiple potential interventions: selective GPR91 modulators targeted to cardiomyocytes, AMPK activators that favor NAD+ biosynthesis, or NAD+ precursors and modulators to replenish intracellular pools. Each approach, however, must be evaluated for tissue specificity, timing, dosing, and long‑term safety given succinate’s pleiotropic roles [3–6]. Key translational questions remain. Will the benefits observed in the “two‑hit” cardiometabolic model generalize across other HFpEF phenotypes (for instance, hypertensive or aging‑related forms)? What is the optimal timing and duration of intervention to capture adaptive windows while avoiding maladaptive chronic stimulation? And can therapies be developed that selectively activate cardiomyocyte GPR91 or downstream AMPK-NAD+ pathways without stimulating harmful signaling in non-myocytes? Addressing these questions will require carefully designed preclinical studies and, ultimately, targeted early‑phase clinical trials.

## Conclusion

Jia et al. [2] deliver an important conceptual advance by showing that succinate can act as a context‑dependent signaling metabolite that, via cardiomyocyte GPR91 and AMPK activation, restores NAD+ homeostasis and ameliorates diastolic dysfunction in cardiometabolic HFpEF. Their work reframes HFpEF as a disorder of disrupted metabolic communication and highlights the succinate-GPR91-AMPK-NAD+ axis as a promising but complex therapeutic target. The challenge ahead is to translate these mechanistic insights into safe, tissue‑targeted interventions that preserve succinate’s cardiomyocyte‑protective signaling while minimizing maladaptive systemic effects, a task that will require precise targeting, rigorous mechanistic dissection, and careful evaluation of timing and dosing.

## Data Availability

No datasets were generated or analysed during the current study.

## Abbreviations

HFpEF: Heart Failure with preserved Ejection Fraction
ROS: Reactive oxygen species
GPR91: G protein-coupled receptor 91 (succinate receptor 1, SUCNR1)
AMPK: AMP-activated protein kinase
CaMKKβ: Calcium/calmodulin-dependent protein kinase kinaseβ
IR: Ischemia-reperfusion
NAD^+^/NADH: Nicotinamide adenine dinucleotide (oxidized/reduced forms)
TCA: Tricarboxylic acid (cycle)
